# Schizophrenia: a tale of two critical periods for prefrontal cortical development

**DOI:** 10.1038/tp.2015.115

**Published:** 2015-08-18

**Authors:** L D Selemon, N Zecevic

**Affiliations:** 1 Department of Neurobiology, Yale University School of Medicine, New Haven, CT, USA; 2 Department of Neuroscience, University of Connecticut Health Center, Farmington, CT, USA

## Abstract

Schizophrenia is a disease of abnormal brain development. Considerable evidence now indicates that environmental factors have a causative role in schizophrenia. Elevated incidence of the disease has been linked to a wide range of disturbances in the prenatal environment and to social factors and drug intake during adolescence. Here we examine neurodevelopment of the prefrontal cortex in the first trimester of gestation and during adolescence to gain further insight into the neurodevelopmental processes that may be vulnerable in schizophrenia. Early embryonic development of the prefrontal cortex is characterized by cell proliferation, including renewal of progenitor cells, generation of early transient cell populations and neurogenesis of subcortical populations. Animal models show that curtailing early gestational cell proliferation produces schizophrenia-like pathology in the prefrontal cortex and mimics key behavioral and cognitive symptoms of the disease. At the other end of the spectrum, elimination of excitatory synapses is the fundamental process occurring during adolescent maturation in the prefrontal cortex. Adverse social situations that elevate stress increase dopamine stimulation of the mesocortical pathway and may lead to exaggerated synaptic pruning during adolescence. In a non-human primate model, dopamine hyperstimulation has been shown to decrease prefrontal pyramidal cell spine density and to be associated with profound cognitive dysfunction. Development of the prefrontal cortex in its earliest stage in gestation and in its final stage in adolescence represents two critical periods of vulnerability for schizophrenia in which cell proliferation and synaptic elimination, respectively, may be influenced by environmental factors.

## Introduction

Almost three decades ago Weinberger^1^ proposed a neurodevelopmental hypothesis for the etiology of schizophrenia. Weinberger's hypothesis synthesized emerging findings from the field of schizophrenia research, attaching particular importance to the observation that symptoms of the disease often surface in adolescence and are associated with stressful life events. Weinberger^1^ suggested that ‘…a fixed ‘lesion' from early in life may interact with normal brain maturational events that occur much later.' This vision of the pathogenesis of schizophrenia was prescient. At the time, the field of neuropathology in schizophrenia was in its infancy. The only evidence for structural brain pathology was the finding of enlarged ventricular volume in patients with schizophrenia.^2, 3^ There was not yet any hard evidence linking early brain insult to schizophrenia, and the concept that the late adolescent maturational process of synaptic elimination might contribute to schizophrenia had only recently been proposed.^4^

In the ensuing years, a wealth of evidence has accumulated from epidemiologic studies to indicate that prenatal and perinatal factors contribute to the risk of developing schizophrenia, leading to virtual universal acceptance of schizophrenia as a neurodevelopmental disease.^5, 6^ Over this same period, neuroimaging studies of schizophrenia subjects have found widespread cortical gray matter loss that is particularly pronounced in certain areas such as the prefrontal cortex,^7^ and neuropathologic examination of the prefrontal cortex has shown that this volumetric loss is due to impoverished neural connectivity rather than loss of neurons.^8^ Taken together, these diverse research findings strengthen the basic tenets of the neurodevelopmental hypothesis: (1) that schizophrenia is a disease associated with early abnormal brain development and (2) that schizophrenia-associated neuropathology, for example, reduction of neuropil in the prefrontal cortex, could be unmasked by the late adolescent process of synaptic pruning.

The notion that environmental factors may have an impact on neurodevelopment and increase the risk of schizophrenia does not repudiate decades of work establishing a robust genetic contribution to the disease.^9^ In this regard, recent genome-wide association studies have estimated the genetic component at 64–81%, with at least 20 genes showing a strong linkage to schizophrenia.^10^ Yet, heritability is still less than 100%, and some of this ‘missing heritability' in schizophrenia could be due to environmental factors.^11, 12^ Notably, studies of the incidence of schizophrenia in twins, although establishing the role of heredity factors in schizophrenia, also provide key evidence for the importance of environmental factors in the disease. The fact that only ~50% of monozygotic twins, who by definition have an identical gene complement, are concordant for the disease argues that differing environmental exposure may trigger the disease in one twin, leaving the other genetically predisposed twin free of illness.^13^ Conversely, shared common environment blurs the distinction in heritability between monozygotic and dizygotic twin pairs; that is, dizygotic twins show greater concordance than would be expected based on genes alone.^14^ With increasing awareness of the importance of both genetic predisposition and environmental factors in the etiology of schizophrenia, some have advanced a ‘two hit hypothesis' such that a predisposing genetic background for schizophrenia comprises the first hit while the second hit is supplied by environmental factors.^15^ Recently, adolescence has emerged as another period of vulnerability to environmental insult that is associated with a higher outcome of schizophrenia and therefore of susceptibility to the second hit.^11^

One of the brain regions most consistently implicated in the pathophysiology of schizophrenia is the dorsolateral prefrontal cortex.^16^ Dysfunction of the dorsolateral prefrontal cortical is thought to be an underlying substrate for thought disorder in schizophrenia.^17^ Patients with schizophrenia perform poorly on an array of tasks that depend on prefrontal cortical function, for example, Continuous Performance (attention), Stroop (cognitive inhibition), Wisconsin Card Sort (cognitive flexibility), Delayed Response (working memory) and N-Back (working memory) tasks.^18^ Moreover, a meta-analysis of magnetic resonance imaging findings has reaffirmed that the dorsolateral prefrontal cortex is a hotspot for gray matter volume deficit in the brains of schizophrenia patients.^19^ Although we fully acknowledge that many other brain regions have been implicated in the neuropathology of schizophrenia, for example, the thalamus, neostriatum, and cerebellum, we have elected to focus on just one area, the dorsolateral prefrontal cortex, in order to comprehensively examine its development and vulnerability to environmental insult.

In this review, we revisit the neurodevelopmental hypothesis, concentrating on two developmental periods, early gestation and adolescence, that have been implicated in the etiology of schizophrenia. Guided by knowledge of basic human neurodevelopment, we identify two developmental processes, cell proliferation in early gestation and synaptic pruning in adolescence, that are prominent during these critical epochs and are essential to normative prefrontal cortical development. We postulate that diverse environmental insults may converge on these processes and perturb normal development of the prefrontal cortex, resulting in a heightened risk of schizophrenia.

## The early prenatal period and schizophrenia

### Maternal infection

Environmental factors that have an impact on prenatal brain development are now known to confer heightened risk for developing schizophrenia in adulthood. One of the earliest links between prenatal environmental perturbation and schizophrenia came from population studies linking the occurrence of influenza epidemics to an increased incidence of schizophrenia in individuals *in utero* at the time of the epidemic.^20^ Subsequent studies documented a heightened risk for schizophrenia in the progeny of flu-exposed populations in Denmark, the United Kingdom and Japan, although a link between influenza exposure and schizophrenia could not be established in several other northern hemisphere populations.^21^ In the southern hemisphere, an association between influenza exposure and schizophrenia was observed for two studied epidemics (1954 A1 and 1957 A2) but not for a third (1959 A2).^22^ In all of the studies with a positive association, the second trimester or midgestation was identified as the period of vulnerability for exposure to influenza. However, one problem with population analyses is that fetal exposure during the epidemic is based solely on presence of the mother in the region of the epidemic without verification of maternal infection.^23^ A later study, using archived maternal blood samples that had been prospectively collected throughout pregnancy to serologically document exposure to influenza virus, found a sevenfold increase in risk for schizophrenia in individuals exposed during the first trimester; no increased risk for schizophrenia was observed for exposure during either the second or third trimesters.^24^

A number of other maternal infections have now been associated with elevated risk for schizophrenia in the progeny. For example, high maternal IgG levels for *Toxoplasma gondii* have been associated with an increased risk of schizophrenia for the offspring.^25^ Serologically verified maternal exposure to rubella in the first trimester of gestation also confers additional risk for schizophrenia spectrum psychosis.^26^ Although the data for herpes simplex virus 2 are not entirely concordant, some studies have established a link between maternal infection and schizophrenia in the offspring.^27, 28^ Notably, very early exposure, during the peri-conceptual phase of pregnancy to genital/reproduction infections also increases the risk for schizophrenia,^29^ as do bacterial infections in the first trimester.^30^ On the other hand, maternal respiratory infection in the second trimester, but not the first or third, has linked to an increase in the incidence of schizophrenia.^31^ Likewise, second-trimester exposure to infectious outbreaks of bronchopneumonia or poliomyelitis has been associated with increased numbers of schizophrenia births.^32, 33^ Thus, maternal infection with a wide range of viral and bacterial agents confers additional risk for developing schizophrenia later in life, and many studies point to the first or second trimester of gestation as critical periods of vulnerability.

### Immune factors

Growing evidence suggests that it is the mother's immune reaction to infection that negatively influences neurodevelopment, ultimately leading to schizophrenia.^34, 35^ For example, the correlation between excess winter/early spring births for schizophrenia and high rates of infectious disorders in the preceding year indicates that it is not the infection *per se* but enduring physiologic changes in the mother that may mediate the effect since infection during the prior winter would have occurred before conception.^36^ A further strong argument for an indirect mechanism stems from the fact that many infectious agents do not cross the placental boundary. Several studies now have established that increased levels of maternal cytokines have a role in increasing risk for schizophrenia.^34, 37, 38^ Along these same lines, linkage between fetal–maternal blood antigen incompatibility and an increase in risk for schizophrenia further implicates immune system reactivity in the etiology of schizophrenia.^39, 40^ Alternatively, maternal inflammation might compromise placental blood flow such that the resulting hypoxia could impair normal brain development.^41^

### Maternal malnutrition

Maternal malnutrition is also a risk factor for schizophrenia. The Dutch Hunger Winter of 1944/1945, because it was a famine with sharply delimited boundaries starting with the imposition of the Nazi blockade in the Netherlands in October 1944 and ending with liberation by the Allied forces in May 1945, afforded an opportunity to study the effects of maternal starvation at different stages in gestation. Individuals conceived at the height of the famine exhibited a twofold increase in the risk for later developing schizophrenia than comparison subjects, whereas maternal famine during midgestation did not increase the risk for schizophrenia.^42^ Subsequent studies of populations undergoing severe famine in China have replicated this finding.^43, 44^ These studies clearly implicate the first trimester of gestation as an important epoch for nutritional solvency and schizophrenia. It should be noted however that deficiencies in individual nutrients, as for example folate and iron, although also associated with an increased risk of schizophrenia, appear to have an impact on gestation in the third trimester.^45, 46^ Abnormally high or low vitamin D levels, as documented by newborn blood samples and therefore unfortunately not linked to a particular prenatal time frame, are also associated with an increased risk of schizophrenia.^47^ Thus, although it is clear that severe maternal malnutrition in the first trimester of pregnancy increases the risk for schizophrenia, the entire picture of how dietary nutrients relate to schizophrenia is less certain.

### Adverse life events

Interestingly, even psychosocial events in the mother's life may contribute to the risk of schizophrenia in her offspring. For example, death or diagnosis of a grave illness in a close relative when occurring during the first trimester of gestation increases the risk for schizophrenia in adult offspring.^48^ Note, however, that an earlier study linked paternal death during mid or late gestation to an increased incidence of schizophrenia, perhaps indicating a wider window for the impact of life stressors.^49^ With regard to the mechanism, it is unclear whether maternal mood is the determining factor. For instance, the effect of unwanted pregnancy was found to have an association with schizophrenia in the offspring by one group but was not replicated by a second.^50, 51^ Further, antenatal depression increases the incidence of schizophrenia in offspring only in those already at genetic risk for the disease.^52^ Some have proposed that stress and ensuing activation of glucocorticoid hormones may be the key factors in mediating the effects of adverse maternal life events on the developing fetus.^53^

In summary, a wide range of prenatal environmental factors have been implicated as risk factors for schizophrenia. Most of these factors impact development during the first two-thirds of gestation. With regard to very early fetal development, an increased risk of schizophrenia has been linked to infection, malnutrition and adverse maternal life events in the first trimester of gestation. This association of schizophrenia with very early neurodevelopment is particularly intriguing, given that human cortical development is barely underway in the first gestational trimester.

## Cortical development in the first trimester of gestation

### Cortical plate formation

**Figure 1:**
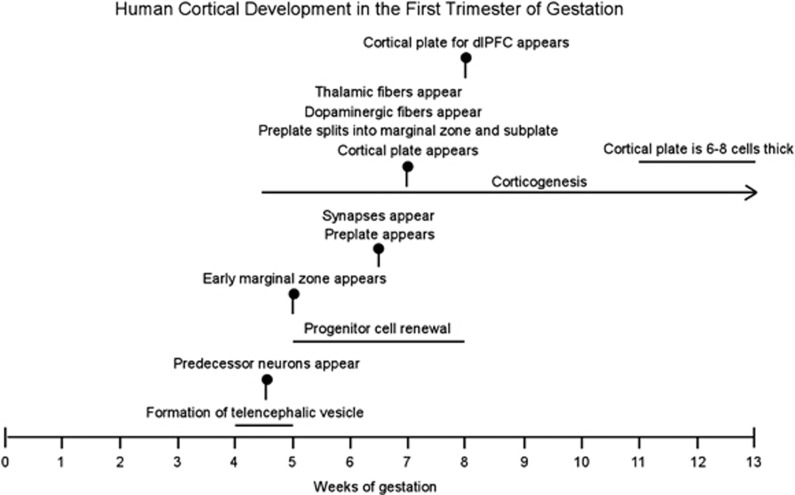
Important milestones in human cortical development are indicated by vertical markers. Ongoing processes are shown as horizontal lines. Note that corticogenesis begins at ~4.5 gw with genesis of preplate neurons: first predecessor cells followed by Cajal–Retzius and other preplate neurons. Corticogenesis of neurons populating the cortical plate commences later, in the 8th gw for the dorsolateral prefrontal cortex (dlPFC). gw, gestational weeks.

Cortical development is a prolonged process in the human fetus (Figure 1). At 5 gestational weeks (gw), the five vesicles of the neural tube have formed, dividing the brain into its major subdivisions: telencephalon, diencephalon, mesencephalon, metencephalon and mylencephalon.^54^ Progenitor cells lining the telencephalic eminence comprise the ventricular zone and, beginning at ~7 gw, these proliferative cells give rise to prospective neurons that form the cortical plate.^55, 56^ Specifically, the ventricular zone and a subsidiary proliferative zone directly subjacent to it, called the subventricular zone, spawn primitive neurons that migrate via contacts with radial glia cells toward the outer margin of the telencephalic vesicle to form the cortical plate.^56^ Later generated neurons migrate past the earlier arriving neurons such that the cortex forms in an inside-out pattern.^57^ Cortical neurogenesis continues throughout midgestation and into the third trimester ending at ~28 gw.^55, 58^ At 13 gw, which is approximately the end of the first trimester, the cortical plate is only six to eight cells thick^59^ such that only neurons destined to form the infragranular layers are present.

Any consideration of how cortical development may be derailed by environmental factors in early gestation must take into account of one glaring fact, that is, half of the first trimester has elapsed before the cortical plate is generated. Moreover, because of the staggered nature of cortical development, cortical plate formation of the most highly differentiated neocortex, including the dorsolateral prefrontal cortex, commences approximately a week later than the estimated date of 7 gw observed for the paleocortex.^59^ Therefore, it may be elucidating to examine what is happening during these first 8 weeks, when the cortical plate destined to form the prefrontal cortex has not yet emerged, in order to understand the potential for perturbed cortical development.

### Progenitor renewal

**Figure 2:**
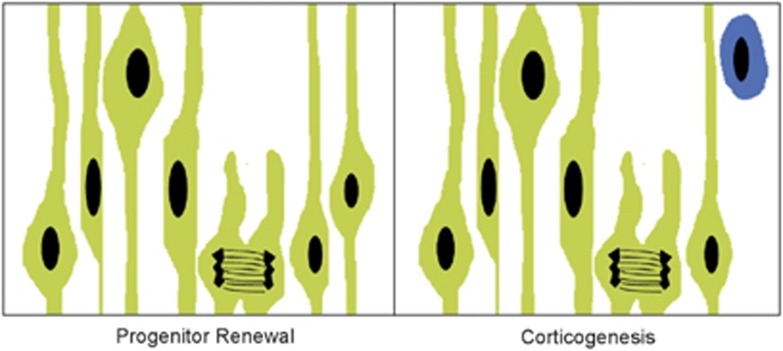
Illustration of symmetric cell division (left) and asymmetric cell division (right) in the ventricular zone of the prospective telencephalon. Progenitor cells (green), also known as radial glial cells, have elongated cell processes that are attached to the ventricular and pial surfaces and form a pseudostratified epithelium. Cell nuclei move toward the pial surface during DNA synthesis and then toward the ventricular surface just before mitosis. In early phases of cortical development, symmetric division gives rise to two identical daughter cells that are both progenitor cells (progenitor renewal). Later in development, asymmetric division (corticogenesis) produces a progenitor cell and a post-mitotic neuron (blue).

Cell proliferation is the most prominent developmental process in the earliest stages of embryonic development, even before generation of the cortical plate has begun. Progenitor cells in the telencephalic ventricular zone undergo two phases of cell division: the first, symmetrical cell division that generates additional progenitor cells and the second asymmetric division that generates prospective cortical neurons (Figure 2).^60, 61, 62^ Symmetric division of progenitor cells enlarges the cortical protomap by adding more progenitors along the tangential axis. Thus, the areal expanse of the cortex is enlarged by the addition of these newly generated proliferative units. In contrast, asymmetric division, or neurogenesis, adds new neurons in a radial direction, generating and increasing the thickness of the cortical plate (Figure 2).^60, 61^ Accordingly, the surface area of the cortical mantle reflects the number of symmetric divisions occurring early in development, as for example a longer period of symmetric cell division in primates results in a much expanded cortical surface relative to that of rodent species.^60, 62, 63^ In humans, intermediate progenitor cells in the subventricular zone form a secondary proliferative site and engage in both symmetric and asymmetric divisions, further enlarging and adding to the complexity of the human cortex relative to that of other species.^64, 65^ Nonetheless, the basic dimensions of the adult cortex in terms of area and thickness are established via progenitor renewal (symmetric division) and neurogenesis (asymmetric division), respectively.

### Preplate formation

During this first 8 weeks of embryonic development, neurons with diverse morphologies and immunoreactivities are generated that predate the existence of the cortical plate proper.^59, 66^ Thus, ‘corticogenesis' in the telencephalic ventricular zone starts with production of these early, largely transient cell populations; corticogenesis of cortical plate neurons begins later at 7–8 gw (Figure 1).^56^ Early generated cells reside in the preplate, a transient layer that forms between the ventricular/subventricular zone and the outer boundary of the telencephalic wall.^56^ Initially, these early generated neurons are evenly spread throughout the entire depth of the preplate but gradually distribute into upper and lower sublaminae.^67^ One of the earliest cell types present in the superficial preplate is the Cajal–Retzius neuron, a cell that expresses reelin, releases this molecule into the surrounding extracellular matrix and is thought to facilitate the inside-out patterning of neuronal disposition in the cortical plate.^68, 69^ Early neurons also are immunoreactive for the calcium-binding proteins, calbindin and calretinin; these include both the superficially located Cajal–Retzius neurons that co-express reelin and more deeply situated cells that are reelin-negative.^59^

With the emergence of the cortical plate, the preplate is split into an overlying marginal zone and underlying subplate.^66, 67, 70, 71^ Recent rodent studies suggest that there are multiple origins for subplate neurons that include extracortical regions of the ventricular epithelium, although this has not yet been established in primates.^72^ In human and non-human primates, the subplate becomes much larger than the cortical plate by the beginning of the third trimester mainly through the addition of ingrowing fibers.^70^ Functionally, subplate neurons mature very early. Subplate neurons exhibit electrical excitability before that of any other cortical neurons (~20 gw), exhibit a rich gene profile and are thought to have an essential role in establishing cortical connectivity.^70, 71, 73, 74^ Although all of the neurons in the marginal zone and the majority of neurons residing in the subplate disappear in early infancy, some subplate neurons survive as interstitial neurons in the white matter subjacent to layer VI.^67, 75^

Recently, neurons born even earlier than Cajal–Retzius cells have been described in the prospective telencephalon.^76, 77^ These ‘predecessor cells' appear soon after neural tube closure, are generated in the diencephalon or basal telencephalon and migrate to take up residence in the preplate of the primordial cerebral cortex.^56, 77^ In contrast, Cajal–Retzius cells and other neurons of the preplate arise locally from the telencephalic ventricular zone, as well as from more distant generative zones in the ganglionic eminences and pallium.^77, 78, 79^

### Subcortical neurogenesis

**Figure 3:**
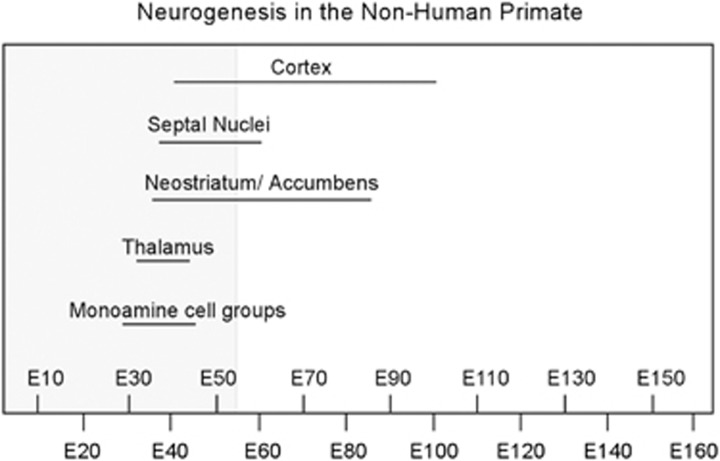
Timeline showing the duration of neurogenesis for various brain structures in the non-human primate as a function of gestational age in embryonic days (E). Neurogenesis occurs in a staggered manner, beginning and ending earlier for subcortical neurons than for cortical neurons. Corticogenesis commences near the end of the first trimester (shaded area) and extends throughout midgestation. Although tritiated thymidine dating of neurogenesis is not possible in the human, morphologic evidence suggests that corticogenesis spans a similar time period in human gestation. Source: on the basis of data from Rakic and colleagues.^57, 80, 81, 82^

Finally, it is important to consider that neurogenesis in subcortical structures precedes that of the cortex (Figure 3).^80, 81, 82^ Therefore, the first trimester of gestation is the period in which neurons in brain regions that are interconnected with the cerebral cortex, for example, the thalamus, striatum and brainstem, are generated. Moreover, two of these subcortical structures, the thalamus and catecholaminergic midbrain nuclei, send afferent input into the cortical primordium at very early stages of development, near the time of emergence of the cortical plate.^76, 83^ Catecholaminergic fibers enter the telencelphalic wall at 7–8 gw, although at this time they are distributed predominantly below the cortical plate in the subplate and intermediate zones, only penetrating the cortical plate at 13 gw.^83^ Early thalamic projections to the cortex also congregate in the subplate zone before entering the cortical plate.^76^ Even at very early stages of development, axons arising from catecholaminergic and thalamic nuclei form synapses with neurons in the subplate, and it is believed that these early transient synapses guide later formation of permanent connectivity within the cortex proper.^70, 73, 84^ From a functional perspective, thalamic afferents also have a role in cortical specification, that is differentiation of the cortical mantle into a mosaic of anatomically and functionally distinct cortical areas.^85^ Whereas intrinsic molecular factors are acknowledged to be the main determinants of cortical specification, disrupted thalamic afferent innervation has been shown to modify the cortical map.^61, 86, 87^

## Cell proliferation: a vulnerable developmental process in early gestation

### Cell proliferation in the first trimester

With respect to cortical development, the first trimester is a period of enormous expansion as the undifferentiated neural tube morphs into a rudimentary cortex. Progenitor cells lining the wall of the prospective telencephalon enlarge the footprint of the cortex,^60^ and progenitor cells in the telencephalon and elsewhere give rise to populations of transient cells that have crucial, if yet not entirely defined, roles in cortical development.^59, 66, 76, 77^ In light of all these generative activities, the most important cortical developmental process occurring in the first trimester certainly is cell proliferation. The cortical plate forms only toward the end of the first trimester, beginning at ~8 gw for the prefrontal cortex, and therefore with respect to the cortex proper, cellular migration, differentiation and establishment of synaptic connectivity all occur later and thus are not major factors in the first trimester. Although cortical neurogenesis does not begin until late in the first trimester, neurogenesis is well underway in brainstem, diencephalic and basal telencephalic nuclei that will eventually form important links in adult cortical neural circuitry. These structures could indirectly influence cortical structure and function via altered afferent input to the cortex or diminished target number for cortical efferents. Indeed, some subcortical structures such as the monoaminergic nuclei and thalamus establish early transient connectivity with the cortical subplate and are thought to have essential roles in guiding the establishment of permanent synaptic connectivity in the cortex.^76, 83^

### Disruption of cell proliferation by environmental and genetic factors

Interference with cell proliferation could represent the final common denominator for a number of environmental and genetic disturbances of embryonic development. For example, cytokines have been implicated in virtually all facets of neurodevelopment, including regulation of neural progenitor cell self-renewal that represents a critical first phase in cortical development.^88^ Thus, it is possible that maternal infection and resulting stimulation of cytokine pathways could disrupt normal mechanisms of symmetric division of neural progenitors in the developing embryo resulting in a smaller cortical surface area.^89, 90^ Elevated glucocorticoid concentrations recently have been shown to interfere with neural progenitor proliferation and neuronal differentiation in hippocampal cell culture, results which suggest that prenatal exposure to maternal stress may also have an impact on cell proliferation.^91^ Certainly, genetic factors involved in controlling neuronal proliferation have been linked to schizophrenia. For example, disrupted in schizophrenia 1 (Disc1) has been shown to have a role in neural progenitor proliferation, suggesting that altered expression of this gene could similarly have an impact on the size of the cortical mantle.^92^ Many other schizophrenia-linked genes also have roles in neurodevelopment, some possibly having an impact on neuronal proliferation.^10^ Recently, a number of genes that are enriched or specific to the mouse subplate also have been identified as risk factors for autism and schizophrenia,^93^ although further studies of these genes in the human subplate are needed to affirm this linkage.

### A primate model of schizophrenia based on disrupted neurogenesis

Over the past two decades, we have examined the anatomical and behavioral effects of reducing neuronal proliferation in early gestation in the non-human primate as a model of schizophrenia. Irradiation via X-ray exposure of the developing fetus in the first trimester (embryonic days 32–40 of a 165-day gestation) has been used to curtail neurogenesis in subcortical structures such as the thalamus that are known to be decreased in volume and have reduced neuron number in schizophrenia.^94, 95, 96^ Fetally irradiated monkeys show reduced thalamic volume and neuron number that is most pronounced in the mediodorsal nucleus and is comparable to that observed in patients with schizophrenia.^97^ In addition, irradiated monkeys have widespread deficits in cortical gray matter volume similar to the pathology observed in schizophrenia patients.^98, 99^ Although the cortical deficits might arise secondary to loss of subcortical afferent and efferent connections, a recent study suggests an alternative explanation: reduction of neuronal progenitor cells.^100^ In the prefrontal cortex of irradiated monkeys, cortical surface area is significantly diminished while cortical thickness is spared, a pattern consistent with a decreased number of cortical proliferative units.^100^ Notably, a similar pattern of selectively reduced cortical surface area in conjunction with normal cortical thickness has been described in first-episode schizophrenia subjects.^101^ Cortical neuron density in the prefrontal cortex of irradiated monkeys is elevated, suggesting a reduction in connectivity,^100^ as has been described in the dorsolateral prefrontal cortex of schizophrenia subjects.^8^ As adults, fetally irradiated monkeys exhibit motor stereotypies, cognitive perseveration and profound working memory deficits, indicating that the prefrontal cortical and corticostriatal loop functions are impaired by fetal exposure to irradiation.^102, 103^ Most intriguingly, the same fetally irradiated animals that exhibit severe cognitive deficits and motor stereotypies as adults were indistinguishable from their normal counterparts as juveniles.^102, 103^ These observations indicate that the ‘lesion' incurred by fetal irradiation in the prefrontal cortex remains dormant and is expressed only as the animals mature to adulthood, perhaps when late adolescent developmental processes unmask the underlying ‘lesion.'

### A rodent model of schizophrenia based on disrupted neurogenesis

Interference with neuronal proliferation is also the basis for a rodent model of schizophrenia in which methylazoxymethanol acetate, a DNA synthesis inhibitor, is administered on gestational day 17 to pregnant rats.^104^ The methylazoxymethanol acetate model reproduces many key features of the schizophrenia phenotype, for example, volume loss in the prefrontal and medial temporal cortices as well as behavioral and cognitive abnormalities.^104, 105^ However, neurodevelopment in the rat at the time of the insult in the methylazoxymethanol acetate model does not correspond to the first trimester of human development; for instance, gestational day 17 in the rat represents the peak of cortical neurogenesis and a time when subcortical neurogenesis is essentially complete. Earlier administration of methylazoxymethanol acetate produces much more severe cortical atrophy and pathology that does not correspond to the more subtle pathology described in schizophrenia.^105^ The global pathology produced by this earlier disruption of neuronal proliferation may reflect the greatly compressed neurodevelopmental timeline in rodents relative to primates, making targeting of specific neuronal populations of neurons more difficult in these species.

## Prefrontal cortical development in adolescence

### Synaptic pruning

**Figure 4:**
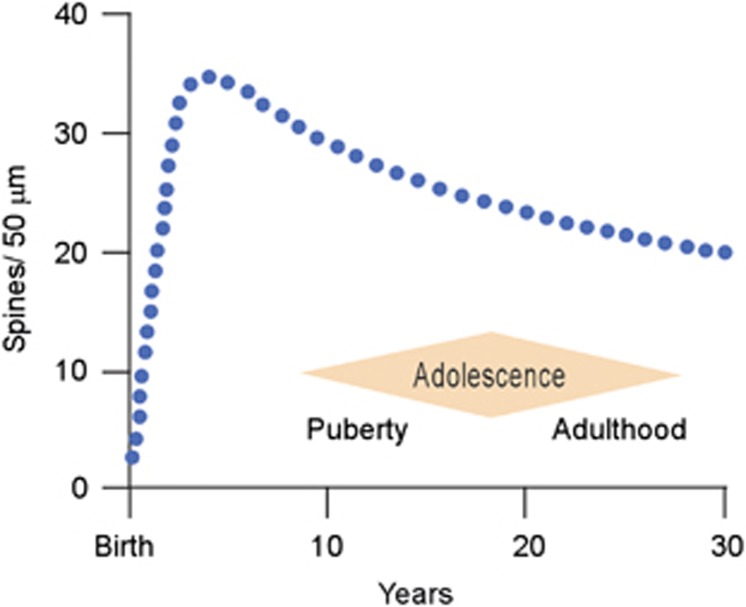
Spine density (blue dotted line) on pyramidal cells in the human dorsolateral prefrontal cortex as a function of postnatal age. Note that there is a net spine loss, indicating pruning of excitatory synapses, beginning in childhood, continuing throughout adolescence, and extending into early adulthood. Adolescence is a period with imprecise boundaries beginning with the onset of puberty and ending at the point in which adult responsibilities are assumed. Source: on the basis of data from apical proximal oblique dendrites in Petanjek *et al.*^109^ and in agreement with Huttenlocher and Dabholkar.^108^

The signature event marking adolescent maturation of the prefrontal cortex in both human and non-human primates is synapse elimination.^106, 107, 108, 109^ Longitudinal magnetic resonance imaging of human subjects has shown that overall cortical gray matter volume peaks before adolescence and then slowly declines until reaching its adult volume.^110^ Studies in human and non-human primates have described a similar pattern for synaptic density, suggesting that adolescence and even early adulthood are periods characterized by reduction of the number of synapses present in the cortex (Figure 4).^106, 107, 108, 109^ Just as the dorsolateral prefrontal cortex is the last region of the cortex to begin generation of the cortical plate, it is also the last cortex to finish the maturational process and achieve the adult state.^108, 110, 111^ Indeed, a recent analysis suggests that the process of synapse reduction in the human prefrontal cortex continues through the third decade of life.^109^ The eliminated synapses are exclusively excitatory contacts,^106, 107^ and therefore synaptic pruning is thought to be critical in establishing the proper excitatory/inhibitory balance in the adult cortex. Inhibitory activity in the prefrontal cortex is an essential component of the network dynamics that underlie cognitive processing and is also the substrate for gamma oscillations that have been associated with cortical computation in many brain areas.^112, 113^ Not surprisingly, disruption of the normal excitatory/inhibitory balance is hypothesized to have a role in psychiatric illnesses, such as schizophrenia.^114^

### Development of executive function

Executive functioning governed by the prefrontal cortex encompasses all the cognitive building blocks of adult rational thinking, for example, attention, internally guided behavior, cognitive flexibility and impulse inhibition.^115^ These cognitive functions exhibit a protracted developmental trajectory that reaches full maturation only in early adulthood,^116^ corresponding with the termination of gray matter volume reduction in the prefrontal cortex.^110^ Therefore, the morphologic changes in synaptic number occurring during adolescence and early adulthood are strongly linked to the emergence of adult executive functioning of the prefrontal cortex. Particularly sharp slopes of gray matter volume decline have been associated with superior intelligence, whereas a delay in reaching peak volume has been associated with attention deficit hyperactivity disorder, a disease in which executive capacity is impaired.^117, 118^

## Schizophrenia, adolescence and environmental factors

### Adolescence and the onset of schizophrenia

In the majority of subjects diagnosed with schizophrenia, the first psychotic break occurs in late adolescence or early adulthood.^119^ Thus, timing of the onset of illness in itself points to the adolescent epoch as a critical period for schizophrenia. Feinberg^4^ was first to suggest that synaptic pruning might be involved in the pathogenesis of schizophrenia. Computer modeling of this process, which has been labeled ‘developmentally reduced synaptic connectivity' has shown that many features of schizophrenia, including symptomatology and age of onset, can be reproduced by exuberant synaptic pruning in adolescence.^120^ Moreover, study of childhood-onset schizophrenia indicates that children diagnosed with this disease have a steeper than normal decline in cortical gray matter volume,^121^ bolstering the link between synapse elimination and schizophrenia. These findings might be interpreted as weakening the association of synaptic pruning with illness onset, as by definition onset in this population occurs before adolescence; however, it is possible that in child onset schizophrenia the developmental lesion is expressed before puberty because this is a particularly severe, rare form of the illness.

### Social factors

It is now well established that postnatal environmental factors related to quality of life modify the risk of schizophrenia. For example, individuals who are raised in an urban environment are more likely to develop schizophrenia than those who grow up in rural environments.^122^ Living in a city during the first 15 years of life increases the risk for schizophrenia in subjects, regardless of their place of residence at the onset of illness.^123^ Whereas most studies have focused on simple population density, some have examined the social characteristics of neighborhoods and have found that social structure may be an important contributor to the added risk. For instance, ethnic homogeneity seems to be a protective factor, whereas conversely minority status in a community is associated with greater risk for schizophrenia.^124, 125^ The latter finding may in part account for the fact that immigrant populations carry a greater risk for the disease.^126, 127^ General socioeconomic status has a less certain relationship to the risk for schizophrenia because these analyses are confounded by the fact that the disability incurred by affliction with schizophrenia leads to diminished social stature.^122^

### Cannabis use

Another factor linked to an increased risk for schizophrenia is cannabis use. Cannabis use, especially if heavy and prolonged, has been associated with persistent psychosis unrelated to cannabis intake and an elevated risk for developing schizophrenia.^128^ Cannabis use in those predisposed to mental illness is associated with higher outcomes of psychosis and schizophreniform illness than in those not at risk.^128^ However, controlled studies show that even in healthy individuals intravenous administration of the principal active ingredient in cannabis can produce transient psychotic symptoms and cognitive deficits that resemble those observed in schizophrenia subjects.^129^ Moreover, growing evidence indicates that outcome is related to the age at which cannabis use commences. For example, one study found that adolescents who engage in cannabis use before the age of 15 may be at greater risk of developing schizophrenic symptoms than those who begin using cannabis after age 15 but before age 18.^130^ These disturbing findings suggest either that early adolescence is a particularly vulnerable time for cannabis-associated insult to the developing brain or that exposure to cannabis throughout adolescence is more deleterious than exposure only during late adolescence. Apart from overt psychosis, adolescent cannabis use has a range of detrimental effects on cognition that may be related to impaired prefrontal cortical function, for example, diminished sustained attention, impulse control and executive functioning.^131^ Animal studies reaffirm the observations in human subjects, demonstrating that administration of cannabinoid substances in adolescence but not adulthood is associated with an array of behavioral and cognitive symptoms akin to those associated with schizophrenia.^132^ Cannabis use in human subjects before age 17 is also associated with smaller whole-brain volumes and smaller percentages of gray matter volume,^133^ perhaps linking cannabis to exuberant loss of synapses during the adolescent period of maturation.

## Dopamine dysregulation: a possible mechanism for environmental disruption of synaptic pruning

### Stress and dopamine

Some studies have indicated that patients with schizophrenia have more stressors in their lives than normal comparison subjects.^134^ Clearly, individuals predisposed to mental illness suffer to a greater degree than non-at-risk subjects from the added burden of a stressful situation, as for example urbanicity.^11^ Lieberman *et al.*^135^ have hypothesized that the onset of schizophrenia during adolescence or early adulthood, which is often associated with stressful life circumstances, might be related to excessive neurochemical stimulation of the same circuits that are targeted by psychostimulants including amphetamines. Thus, dopamine hyperstimulation may be a common mediator for the stress effects on prefrontal cortical development that increase the risk for schizophrenia.

It has been postulated that the underlying factor in many psychosocial adversities linked to schizophrenia, for example, minority status, is social defeat and that heightened sensitivity of mesolimbic dopamine circuits may be the biologic consequence.^136^ Perhaps overlooked is the mesocortical projection, which is also important in conferring emotional valence to both rewarding and aversive stimuli and is important in mediating the effects of stress on prefrontal cortical function.^137, 138^ Dopamine acting via the D1 receptor enhances memory field activity in the prefrontal cortex when present at an optimal concentration, whereas too much or too little dopamine degrades network activity.^139^ Stress overloads the prefrontal cortex with dopamine, thereby producing a state that is detrimental to executive functioning mediated by the prefrontal cortex.^137^ Dopamine stimulation of the D1 receptor also is a major modulator of synaptic plasticity in the prefrontal cortex and therefore may have a role in shaping synaptic connectivity.^140^ As has been shown for cognitive functioning, there is an optimal level of dopamine stimulation for long-term potentiation in the prefrontal cortex.^141^ Importantly, long-term potentiation and long-term depression mechanisms not only strengthen or weaken synaptic connectivity, these processes ultimately facilitate the generation or elimination of synapses.^142, 143^ Because stress induces a long-lasting inhibition of long-term potentiation mechanisms in the prefrontal cortex,^141^ stress could reduce synaptic number by tipping the balance toward long-term depression. One possibility is that long-term depression mechanisms are accentuated during the period of adolescence when synaptic elimination is prominent.^144^ If so, elevated dopamine stimulation of the prefrontal cortex in response to stress could have a more potent effect on synaptic plasticity and connectivity during the adolescent period, perhaps resulting in exuberant synaptic pruning.

### Cannabis and dopamine

Whether the link between cannabis and schizophrenia also involves altered dopamine neurotransmission is a matter for speculation at this point. Cannabis and dopamine are known to interact in the mesolimbic and mesocortical brain reward systems, including the ventral tegmental area, nucleus accumbens and medial prefrontal cortex.^138^ As the cannabinoid receptor CB1 is widely distributed throughout the forebrain,^145^ the possibility of a similar interplay between cannabinoid stimulants and dopamine is possible anywhere that dopamine receptors exist, as for example in the dorsolateral prefrontal cortex. Although this potential interaction has not been studied extensively, some evidence points to such an association. For example, a mouse knockout of the catechol-O-methyltransferase gene, an essential enzyme for degradation of dopamine in the prefrontal cortex, exhibits more pronounced schizophrenia-like phenotypes, including spatial working memory deficits, following adolescent administration of cannabinoids compared with the wild-type mouse.^146^ This study supports the hypothesis that modification of dopamine function, in this case via genetic intervention but in real life circumstances in response to stress, can intensify the effects of cannabis use in adolescence and cause long-term changes in prefrontal cortical function. Whether the inverse is true, that is, that cannabis intake can modify prefrontal cortical dopamine neurotransmission with similar long-term consequences, is not clear.

### Animal models

Animal studies have begun to address the question of whether stress, and in particular the dopamine surge brought on by stress, can alter prefrontal cortical connectivity. It is well established that prolonged exposure to stressful conditions reduces spine density in the prefrontal cortex.^147, 148^ Studies in the non-human primate have shown that intermittent, escalating doses of the dopamine stimulant amphetamine produce profound and long-lasting cognitive dysfunction that is accompanied by spinodendritic atrophy especially in supragranular layers of the prefrontal cortex and that both cognitive and morphologic deficits are reversed by long-term treatment with a D1 antagonist.^149, 150^ These studies support the hypothesis that dopamine acting via the D1 receptor may be the mediator for stress-induced changes in spine density. The primates in these studies were adults at the time of the treatment; therefore, we can only speculate that comparable amphetamine sensitization in adolescence might have more deleterious effects on spine density and cognition. Likewise, in human subjects with a genetic predisposition to schizophrenia, heightened dopamine stimulation in response to stress during adolescence could have a profound effect on prefrontal connectivity and function.

## Conclusions

**Figure 5:**
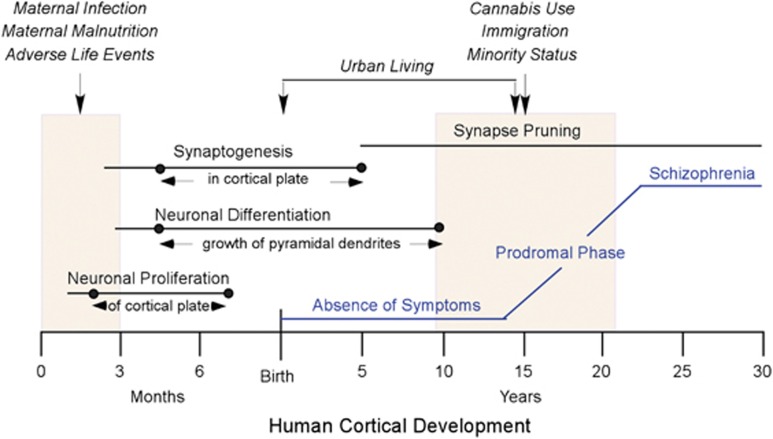
Summary showing human cortical development, environmental factors that increase the risk for later development of schizophrenia and clinical progression of the disease. Major processes in prenatal cortical development (in months) and postnatal cortical development (in years) are indicated by horizontal lines. Dots on the lines indicate the extent of those processes as they occur in the cortex proper, as detailed in text between arrows. Shaded boxes indicate the first trimester of gestation and adolescence. Above the shaded boxes, environmental factors (in italics) that may have an impact on neural development and increase the risk of developing schizophrenia are shown (vertical arrows). The clinical course of schizophrenia from an asymptomatic state in childhood to full expression of the disease in late adolescence/early adulthood is shown in blue.

The early prenatal period and adolescence are critical periods for development of the prefrontal cortex (Figure 5). The first trimester of gestation represents a period of growth in which proliferation of cortical progenitors, preplate neurons, predecessor cells and subcortical neurons is prominent. At the other end of the spectrum, adolescence marks the final stage of prefrontal cortical development in which excitatory synapses are pruned to generate the proper excitatory/inhibitory balance in the cortex. A wide spectrum of environmental factors impinging on the brain during these critical periods can modify the trajectory for prefrontal cortical development and shift the balance toward mental illnesses such as schizophrenia. Animal models are helping to identify the underlying mechanisms that may be influenced by these environmental disturbances in an effort to more fully understand the etiology of schizophrenia.
